# Progressive cutaneous viral pigmented plaques in three Hungarian Vizslas and the response of lesions to topical tigilanol tiglate gel

**DOI:** 10.1002/vms3.85

**Published:** 2017-11-10

**Authors:** Naomi Hansen, Nikianna Nicholas, Graeme Pack, John T. Mackie, Michael Shipstone, John S. Munday, Paul Reddell, Geoff Orbell, Richard Malik

**Affiliations:** ^1^ Greencross Noosa Veterinary Clinic Tewantin Queensland Australia; ^2^ Nikianna Nicholas Medivet Kingston Surrey UK; ^3^ Purton Vets Purton, Swindon UK; ^4^ QML Pathology Arndale Shopping Centre Springwood Queensland Australia; ^5^ Veterinary Specialist Services Unit 14 Springwood Centre Underwood Queensland Australia; ^6^ Institute of Veterinary, Animal and Biomedical Sciences Massey University Palmerston North New Zealand; ^7^ QBiotics Ltd Yungaburra Queensland Australia; ^8^ New Zealand Veterinary Pathology Institute of Veterinary Animal and Biomedical Sciences Massey University Palmerston North New Zealand; ^9^ Centre for Veterinary Education University of Sydney Sydney New South Wales Australia

**Keywords:** papillomavirus, dog, Vizsla, papillomatosis, pigmented plaques, skin

## Abstract

Cutaneous pigmented viral plaques is a disorder of epidermal growth caused by canine papillomavirus type 4 (CPV‐4). There is currently no standard of care for managing this condition and it has not been reported in the Hungarian Vizsla. This case series documents the clinical features of canine pigmented viral plaques in Hungarian Vizsla dogs and the treatment of a severe case using a novel topical agent tigilanol tiglate (EBC‐46). A 4‐year‐old spayed Hungarian Vizsla in Australia was presented for multiple cutaneous pigmented plaques extending from the ventral cervical region. Lesions were neither painful nor pruritic. The number and size of these sessile plaques increased over time, with the largest lesions eventually taking on an exophytic (wart‐like) appearance. These lesions did not affect the dog's wellbeing. Two much less severe cases in a 5‐year‐old Vizsla from the UK and a 7‐year‐old Vizsla from New Zealand were also diagnosed. Histology was consistent with papillomavirus‐induced pigmented plaques and CPV‐4 DNA sequences were amplified from paraffin‐embedded formalin‐fixed tissue using the polymerase chain reaction from the most severely affected patient. Topical imiquimod was ineffective although used for only a short time. Two topical applications of novel anti‐neoplastic diterpene ester tigilanol tiglate as a gel, 9 days apart, greatly reduced the size and number of lesions in a limited portion of skin treated, over the lateral hock. While CPV‐4 has been previously reported to cause pigmented plaques, most commonly on pug dogs, but sporadically on other breeds, this is the first report of this virus causing plaques in Hungarian Vizslas. The cases illustrate some of the difficulties in diagnosing papillomavirus‐induced disease in dogs, especially in its early stages. Topical tigilanol tiglate is a potentially useful topical therapy for this viral‐induced disorder of cell growth and represents a treatment deserving of further investigation.

## Introduction

The term ‘canine pigmented plaques’ refers to a condition seen predominantly in pugs and miniature schnauzers caused by a canine papillomavirus (Nagata *et al*. 1995; Nagata & Rosenkrantz 2013). In these two breeds, the predisposition is thought to be inherited as an autosomal dominant trait, presumably as a result of a genetically programmed immunodeficiency affecting innate or cell‐mediated immunity (CMI) (Stokking *et al*. 2004). Dogs with other causes for immune suppression can also develop the condition (Le Net *et al*. 1997). Lesions consist of multiple, scaly, pigmented macules, plaques and sometimes papules, commonly situated on the ventral neck, ventral trunk, abdomen and extremities. Canine pigmented plaques progress over time (i.e. do not regress), a behaviour distinct from other canine papillomavirus‐associated dermatoses (Nagata *et al*. 1995; Nagata & Rosenkrantz 2013). There is potential for malignant transformation to squamous cell carcinoma (Nagata & Rosenkrantz 2013). The familial nature of this disorder suggests it may be equivalent to the human condition epidermodysplasia verruciformis, a genetically determined susceptibility to certain human papillomavirus (PV) infections (Nagata *et al*. 1995.)

This paper describes canine pigmented plaques in three Hungarian Vizslas, one living in Australia and the second and third domiciled in the United Kingdom and New Zealand, respectively, and the use of a topical gel formulation of the novel anti‐neoplastic agent tigilanol tiglate (QBiotics) as therapy in one of these patients. The first case was especially interesting because of the atypically severe nature of its lesions.

## Case Reports

### Case 1

A 4‐year‐old spayed Hungarian Vizsla living on the Sunshine Coast, Australia was first noted to have skin lesions in 2010. Lesions were recorded as an incidental finding on physical examination during an annual health check. According to the patient's records, the problem started as numerous pigmented areas on the skin of the ventral cervical region. Lesions developed a crusty surface and then increased in number and size, and extended their distribution ventrally towards the sternum over 7 to 8 months. Some lesions lost their crusty surface and persisted as smooth, variably pigmented plaques. The epidermal proliferations did not adversely impact the dog in any way. Specifically, they were neither painful nor pruritic.

Four biopsies were obtained using a skin biopsy punch (3 mm diameter) in December 2010 when the dog was 5 years old. These samples were submitted to a veterinary laboratory (not associated with any of the current authors) for histological assessment. Microscopic examination revealed mild to moderate orthokeratotic hyperkeratosis of the epidermis, acanthosis and basaloid hyperplasia with scattered apoptotic keratinocytes. Two sections revealed patchy epidermal dysplasia. The superficial to mid‐dermis was variably oedematous with a mild, perivascular inflammatory cell infiltrate dominated by mast cells and eosinophils, with small numbers of lymphocytes and plasma cells. No definitive diagnosis was offered, although allergic dermatitis confounded by a solar dermatitis was suggested as a possibility.

**Figure 1:**
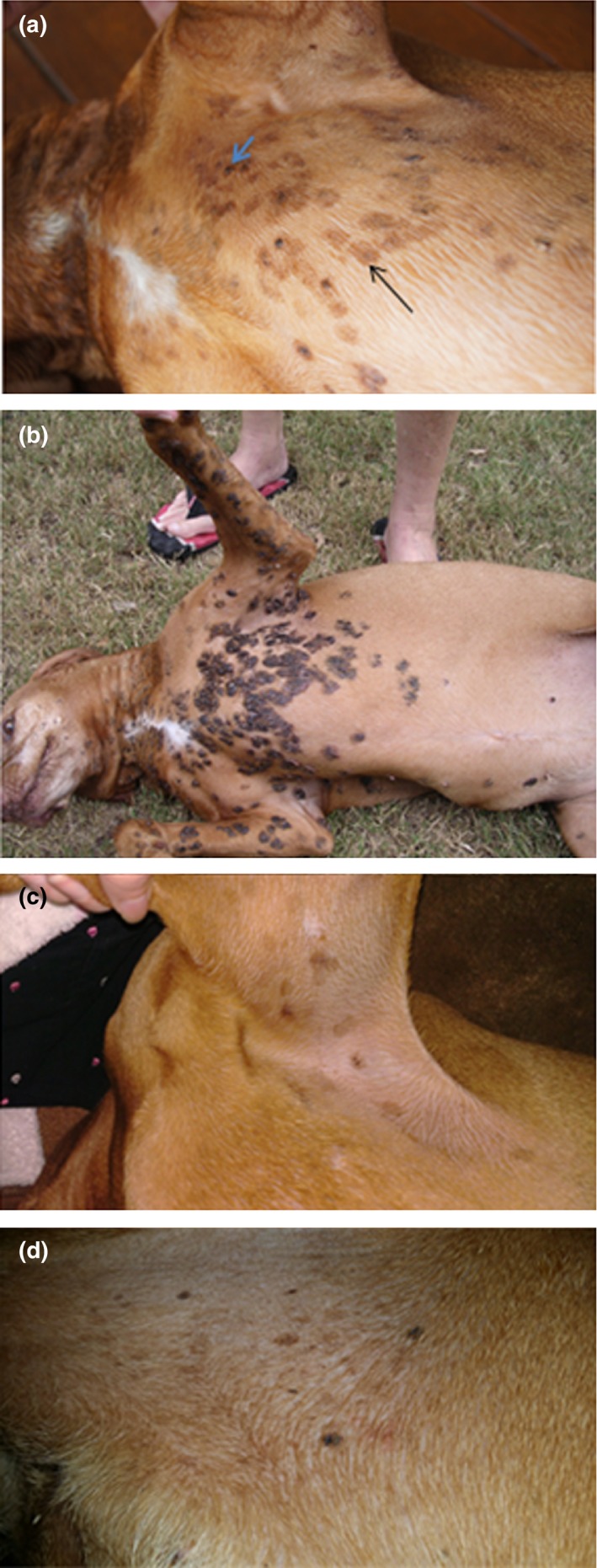
Appearance of pigmented papillomas on Case 1, 2 & 3. (a) shows lesions in Case 1 in 2011, while the lesions observed in 2015 are illustrated in (b). Comparing (a & b) provides an appreciation of the slow but insidious progression of lesions over time. In (a), small pigmented plaques (3–8 mm diameter) on the skin of the sternum and ventral cervical region are highlighted by a narrow black arrow in one instance; approximately 10% of the lesions appeared exophytic (e.g. blue arrow), consisting of dark grey pigmented proliferations. (c) shows representative lesions from Case 2 in 2014, while (d) illustrates lesions from Case 3. Note Case 1 (a & b) is much more severely affected than Cases 2 (c) & 3 (d).

The dog was referred to a veterinary dermatologist (MS) in February 2011. On examination, several small pigmented plaques (3–8 mm diameter) were noted on the skin of the sternum and ventral cervical region. Approximately 10% of lesions appeared exophytic, consisting of dark grey pigmented proliferations (Fig. 1a). The dog was re‐examined in May 2011, with some improvement noted, as some of the lesions had apparently resolved. The lesions were not causing the dog any discomfort, so therapeutic interventions were not implemented.

The dog was examined again in September 2011 because existing lesions had increased in size and several new lesions had developed over the dog's head. Lesions were reported to be pigmented exophytic plaques (3–6 mm in diameter) on the sternum, axilla and ventral abdomen. Five additional punch biopsies (6 mm diameter) were obtained at this time from the skin of the ventral chest. One sample was taken from a new lesion, while four were taken from lesions showing prominent pigmentation that had been present over an extended period.

Examination of the biopsy specimens revealed similar, albeit more marked changes to those observed in earlier biopsies. The most significant changes were within the epidermis. Moderate to marked hyperplasia was visible with a thickened epidermis, forming short exophytic folds. The thickened epidermis was covered by increased loose keratin. A feature within all the biopsy specimens was a prominence of keratinohyalin granules within the epidermis. Epidermal hyperplasia extended into hair follicles. Minimal dysplasia was visible within the epidermis and no cytological evidence of papillomavirus infection (such as cells with blue‐grey granular cytoplasm of koilocytosis) was visible. Large quantities of pigment, likely melanin, were visible within the epidermis as well as within the superficial dermis underlying the lesions. A mild increase in lymphocytes and plasma cells was observed in the dermis. The presence of multifocal epidermal hyperplasia suggested the possibility of canine papillomavirus infection. Immunohistochemistry to detect papillomavirus antigen was performed using rabbit anti‐bovine papillomavirus type 1 (BPV‐1) antibodies (Histology Laboratory, School of Veterinary and Life Sciences, Murdoch University). Immunostaining was visible within positive controls, but no immunostaining was visible within the samples.

Despite negative immunochemistry, the histological and clinical features were supportive of viral papillomatosis, so the patient was treated using imiquimod cream (every 2nd day for 4 days, then daily for 5 days; Aldara, 3M), a drug useful in the management of some human papillomavirus infections. Imiquimod was applied to lesions after first removing the superficial sebum/lipid layer overlying the epidermis with methylated spirits. This treatment did not reduce the size or number of the plaques over a 10‐day trial period.

**Figure 2:**
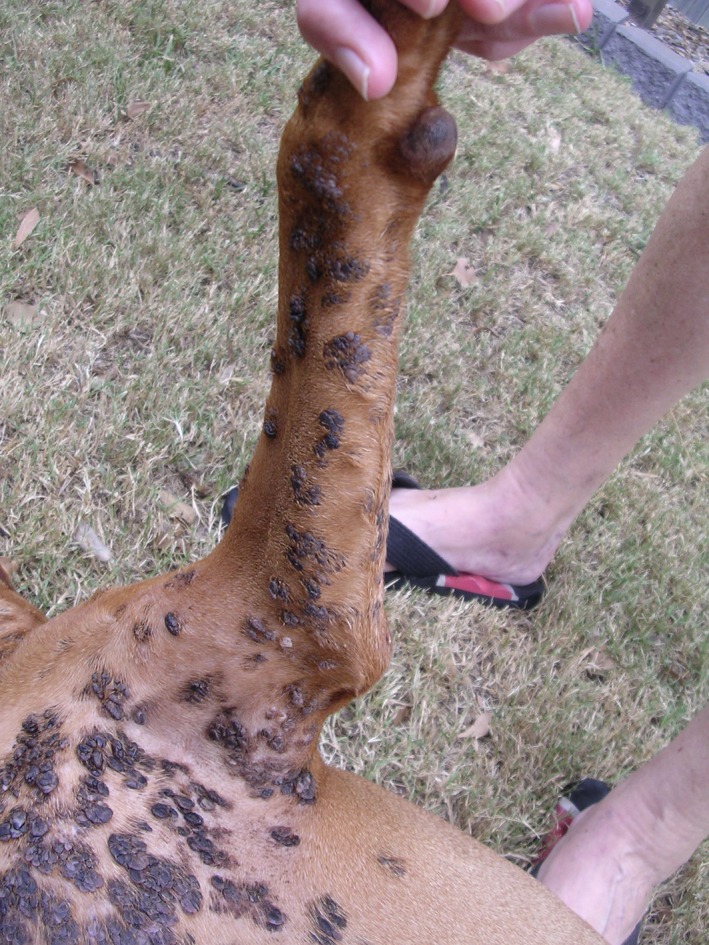
Close up of the lesions from Case 1 in 2015, to demonstrate their heavy pigmentation and exophytic nature of the lesions.

**Figure 3:**
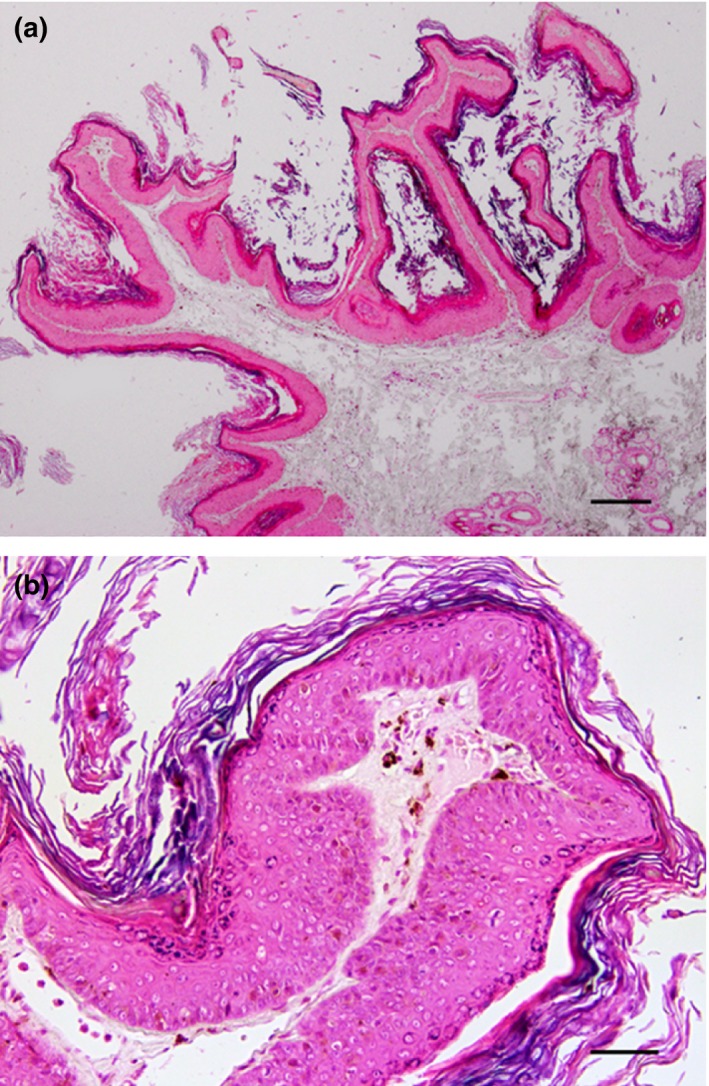
Histology of the skin biopsy from fully developed lesions. (a) The pigmented plaque appears as an exophytic focus consisting of folded hyperplastic epidermis that is covered by increased quantities of dense keratin (H&E, bar = 175 *μ*m). (b) Moderate hyperplasia of the epidermis is visible with increased quantities of melanin pigment both within the epidermis and within the underlying dermis. No evidence of papillomavirus‐induced cytopathic changes is present (H&E, bar = 45 *μ*m).

In March 2015, the dog was presented for routine vaccination. Physical examination revealed numerous pigmented cutaneous plaques on the ventral cervical region and sternum, extending to both axillae (Figs. 1b and Fig. 2). Representative lesions were biopsied under local anaesthetic and samples were again submitted for histology (JTM). All sections (Fig. 3a,b) exhibited similar findings to those observed in earlier biopsies. A diagnosis of multiple pigmented plaques was made. The polymerase chain reaction (PCR) was used to investigate if papillomavirus (PV) DNA was present within the samples. Briefly, total genomic DNA was extracted from shavings of paraffin‐embedded formalin‐fixed tissue (PEFF) as described previously (Munday *et al*. 2007). To amplify PV DNA, both FAP59/64 primers, designed to detect a wide range of human cutaneous PV types and the MY09/11 consensus primers, designed to detect a wide range of human mucosal PV types, were used as described (Waropastrakul *et al*. 2012). DNA from a canine oral papilloma and a bovine fibropapilloma were positive controls for the FAP59/64 and MY09/11 primers, respectively, while no template DNA was added to the negative control. Only the MY09/11 consensus primers produced an amplicon of the expected length which was then purified, sequenced and compared to sequences within GenBank, as described (Munday *et al*. 2007). The amplicon sequence was identical to that of canine papillomavirus type 4 (CPV‐4).

**Figure 4:**
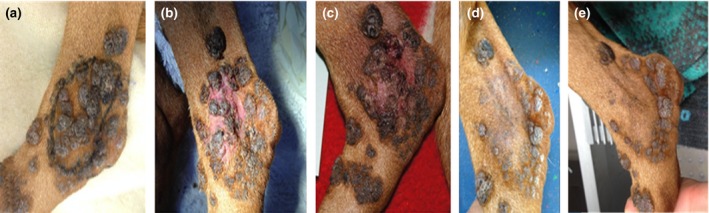
The effect of topical EBC‐46 gel on a representative number of CPV4‐induced canine papillomas and pigmented plaques over the lateral hock of Case 1. The area to be treated (approximately 5 cm by 3 cm) was delineated using a marker pen (a), and then a thin layer of EBC‐46 gel (0.3 mL in total) was applied sparingly over this region, with the dog restrained such that it could not lick the formulation off the skin for 15 min. The treated area became warm and inflamed soon after application of the gel, and after 7 days the exophytic lesions were obviously reduced in size (b). The appearance 1 day after a second application of the gel (9 days after the first topical application) is illustrated in (c), while the appearance of the treated area 2 weeks after the second EBC‐46 application is provided in (d). The appearance of the area over the lateral hock treated with EBC‐46 4 weeks later is shown in (e); note the near complete elimination of the pigmented exophytic lesions.

The effect of tigilanol tiglate gel (QBiotics) on representative lesions was determined by applying 0.3 mL of a topical gel formulation (5 mg/mL) over a small oval portion (3 × 5 cm) of affected skin on the lateral hock on November 3rd 2015 as a single application. The drug was supplied at no cost by the manufacturer. The target area was delineated with a marker pen (Fig. 4a). The treated area became inflamed over several hours. At the 7 day re‐examination, there was a reduction in both the size and proliferative nature of the lesions (Fig. 4b) within the treated area. A second application was made 9 days after the first application (November 12th), by which time all inflammation had resolved. The second application was identical in dose to the first but produced a more marked inflammatory response, with mild discomfort in the area treated (Fig. 4c). Inflammation resolved after 3 days and there was an unequivocal improvement by day 6: many lesions had resolved, with a reduction in size of persisting lesions. Importantly, there were no detrimental effects to the apparently unaffected skin between the exophytic lesions (Fig. 4d; photo taken December 10th 2015). By 4 weeks after the second treatment, there was almost complete resolution of the lesions in the treated area (Fig. 4e; photo taken January 5th 2016). Despite the favourable response, the dog's owner did not wish to treat additional portions of affected skin because of the extent of the remaining lesions, their apparently benign nature and the discomfort likely to be experienced as a result of topical therapy. The dog subsequently developed two trichoepitheliomas on lesioned skin on the dorsal thoracic area; these benign tumours were removed by marginal resection, examined histologically, removed with clear margins and have not recurred at the time of writing.

### Case 2

A 5‐year‐old spayed Vizsla domiciled in the United Kingdom was presented in November 2013 for assessment of numerous pigmented skin lesions on the limbs and ventrum first noted several months previously (Fig. 1c). According to the owner, the condition did not appear to be progressing and skin changes were not causing discomfort. Lesions were variably pigmented, grey/brown in colour and plaque‐like. Two lesions were exophytic and crusting was noted on the surface of two other lesions.

Four skin biopsies were obtained using a skin biopsy punch (6 mm diameter) and submitted to a veterinary laboratory. Histology was consistent with pigmented viral plaques. Dysplastic changes were identified in one section, which raised concerns for potential progression to squamous cell carcinoma (SCC). Routine haematology and biochemistry was unremarkable. The serum thyroxine (T4) concentration (4.4 nmol/L) was less than the canine reference interval (RI; 13–51 nmol/L), the serum TSH (0.25 ng/mL) was within the RI (0.00–0.50 ng/mL) and the free T4 by equilibrium dialysis was normal (7.1 pmol/L; RI 6–40); it was reasoned that the low T4 concentration was due to non‐thyroidal illness or normal for Vizslas (Thuroczy *et al*. 2007). Potential cause(s) for systemic immunosuppression were not identified.

The dog was treated with azithromycin (10 mg/kg orally once daily for 10 days), which had no effect on the size or the number of lesions. Due to further progression noted by the owner in December 2013, multiple lesions were removed using a skin biopsy punch in January 2014. Lesion distribution was again over the limbs and ventrum. Biopsy specimens were submitted to the same laboratory and again confirmed as being consistent with pigmented viral plaques. Attempts to amplify PV nucleic acid from PEFF tissues specimens from these biopsies were unsuccessful.

Due to the presence of numerous lesions, continued surgical removal was not considered a viable long‐term option. The owner elected to monitor the patient and only remove lesions where progression raised the concern for transformation to SCC. Topical treatment with imiquimod was discussed but declined. Despite an initial spontaneous reduction in lesion numbers documented in the clinical records (May 2014), further lesion progression was noted (July 2014). A further four lesions were removed in July 2014 and a further single lesion was biopsied in January 2015. Histological assessment of all lesions was consistent with pigmented viral plaques.

### Case 3

A 7‐year‐old spayed Vizsla domiciled in New Zealand was presented in October 2016 to investigate six non‐progressive pigmented skin lesions on the limbs, axillae and ventrum of at least 4 months duration. Lesions were plaque‐like with adherent scale and brown‐black pigmentation and were well circumscribed from surrounding normal skin (Fig. 1d).

Excisional biopsies were taken of four lesions and submitted to a veterinary laboratory for histology. All lesions had a similar microscopic appearance characterised by locally extensive papillary epidermal hyperplasia with moderate basket‐weave orthokeratotic hyperkeratosis. Diffusely, the epidermis was hyperpigmented and contained markedly increased numbers of keratohyalin granules in the stratum granulosum, with marked variation in size. No dysplastic changes were identified in any of the masses examined. The owner elected to monitor the remaining masses for evidence of progressive behaviour. No topical treatment was administered.

## Discussion

Pigmented plaques are uncommon skin lesions induced by papillomaviruses (PVs) (Nagata *et al*. 1995; Le Net *et al*. 1997; Stokking *et al*. 2004; Nagata & Rosenkrantz 2013). PVs are epitheliotropic and have high species and location specificity. Due to their ability to influence cell growth and replication, PVs are established causes of both hyperplastic (warts or papillomas) and neoplastic lesions of the skin and mucous membranes of humans and animals (Munday *et al*. 2011). In dogs, papillomaviruses have been associated with both oral and cutaneous papillomas and pigmented plaques. A total of 17 canine papillomaviruses have been fully sequenced to date, including many that have been amplified from pigmented plaques (Le Net *et al*. 1997, Nagata *et al*. 1995, Stokking *et al*. 2004; Munday *et al*. 2007, 2011; Lange *et al*. 2011; Luff *et al*. 2012; Waropastrakul *et al*. 2012; Nagata & Rosenkrantz 2013). While CPV‐4 has been reported to cause pigmented plaques, typically in pugs, this is the first report of CPV‐4 in a Vizsla. Pedigrees were available for the first two cases, which did not appear to be closely related (data not presented). This is the first report of CPV‐4 from any dog from Australia, although as papillomaviruses typically have a worldwide distribution, the detection of this papillomavirus in Australia was not unexpected.

Pigmented plaques classically present as multiple, dark, plaque‐like, hyperkeratotic lesions that range in size, but generally remain less than 3 cm in diameter. Plaques are usually flat to slightly raised and located in clusters on the limbs, axillae or abdomen. Unlike classic exophytic papillomas (e.g. oral papillomatosis of young dogs) or endophytic papillomas (e.g. inverted papillomas), canine pigmented plaques tend to occur in older dogs and do not to spontaneously regress. All nine PVs (CPV‐3,‐4,‐5, ‐8, ‐9, ‐10, ‐11, ‐12, ‐14, and ‐16) that have been amplified from pigmented plaques in dogs belong to the Chi‐papillomavirus genus (Rector & Van Ranst 2013; Luff *et al*. 2015).

The main clinical significance of PV‐induced pigmented plaques is their potential to progress to *in situ* or invasive carcinomas (Munday & Kiupel 2010). CPV‐3 has been associated with pigmented plaques that progressed to *in situ* and invasive SCC in both immunocompetent and immunocompromised dogs (Munday & Kiupel 2010). This CPV‐3‐associated malignant transformation occurred over a 20 month to 5‐year period (Munday *et al*. 2011). Although CPV‐4 is closely related to CPV‐3 phylogenetically, unlike CPV‐3, malignant transformation has not been associated with CPV‐4 in the pug or in the other breeds in which CPV‐4‐induced disease has been reported (Luff *et al*. 2012). Lesions in the present cases appear slowly progressive, with no evidence of neoplastic transformation observed over 5 years (Case 1), 2 years (Case 2) and 1 year (Case 3). The presence of dysplastic features in some biopsies (as in Case 2) may, however, remain a cause for concern. As there appears to be a difference in behaviour between CPV‐3 and CPV‐4‐induced pigmented plaques in dogs, determination of the causative PV type by genomic sequencing following PCR amplification of viral nucleic acid helps predict the likely progression of lesions.

Pigmented plaques associated with CPV‐4 are most commonly reported in pugs with proposed autosomal‐dominant inheritance (Nagata *et al*. 1995; Nagata & Rosenkrantz 2013). Affected dogs are suspected of having an underlying immunodeficiency, possibly involving innate or cell‐mediated immunity, which permits PV to persist unchecked. The documentation of CPV‐4 infection in three Vizslas suggests this breed may also have an underlying immunodeficiency, although none of the affected dogs demonstrated any other clinical features consistent with immune dysfunction. It is likely that all three dogs had an inherent inability to mount an appropriate immune response to the CPV‐4. Infection with CPV‐4 has also been reported sporadically in a pointer (left hind limb), Chihuahua (ventral thorax and right inguinal region), bulldog (abdomen), Wheaton terrier (left and right hind limbs), and Australian terrier (base of tail) (Luff *et al*. 2012).

In the index case (Case 1), a diagnosis of pigmented plaques was not made from the initial histological examination. Early PV lesions typically contain mild epidermal hyperplasia and can be difficult to distinguish from other causes of epidermal thickening. Features that can be helpful include the presence of large quantities of clumped keratohyalin granules within the epidermis and extension of epidermal hyperplasia into hair follicles, as observed in Case 1). Self‐trauma is a common cause of epidermal hyperplasia that can be differentiated from PV‐induced hyperplasia by the presence of loose keratin overlying PV‐induced lesions.

Immunohistochemistry for PV antigens failed to confirm a PV aetiology in this case. Negative immunostaining could have been due to two causes. Firstly, it is uncertain whether the anti‐BPV‐1 antibodies used by the Perth laboratory cross‐react with CPV‐4 antigen. Secondly, the antibodies target PV L1 protein, which is only expressed late within the papillomaviral life cycle; thus, if no active viral replication is present within the lesion, then no immunostaining is evident. In the experience of one of the authors (JSM), few PV‐induced lesions contain PV L1 antigen in the absence of PV‐induced cytopathology. A PV aetiology in Case 1 was confirmed by identifying PV DNA within PEFF tissue. As PCR is not reliant on PV protein expression, this technique has a much higher sensitivity for detecting a PV infection than immunohistochemistry (IHC).

In the three cases reported here, one or more of four different treatments were provided to the patients: (1) simple surgical excision, (2) topical imiquimod cream, (3) oral azithromycin and (4) topical therapy with the novel anti‐neoplastic agent EBC‐46 (a diterpene ester which activates protein kinase C). Imiquimod, a Toll‐like receptor‐7 agonist, is thought to stimulate the patient's own immune system (Gill *et al*. 2008); the drug itself has no *in vitro* activity against PV, but stimulates monocytes and macrophages to release cytokines that can induce regression of some PV‐induced lesions by generating a Th1 ‘cytokine milieu’. Imiquimod was trialled in Case 1, but proved to be without benefit, although it was only trialled for 10 days and lesions may take up to 4 weeks to respond according to limited information published. In Case 2, imiquimod was refused by the owners, who instead elected to trial azithromycin on the basis of a single report alleging efficacy for treating canine papillomavirus lesions (Yağci *et al*. 2008). This antimicrobial agent, however, had no impact on the lesions and the use of azithromycin in this setting has subsequently been discredited.

The novel anti‐neoplastic agent tigilanol tiglate (Boyle *et al*. 2014; Lickliter *et al*. 2015) was trialled in Case 1 and proved highly effective. Two applications of a gel formulation were applied topically, with a period of 9 days between applications. The treatment caused local inflammation which appeared to affect both the cauliflower‐like pigmented lesions and interspersed normal skin. This was associated with limited local discomfort, but no systemic malaise. The first application had a clear impact on the lesions. The second application seemed even more effective and within 21 days the treated portion of skin was cleared of warty lesions, with restoration of normal dermal pigmentation and regrowth of normal fur. Tigilanol tiglate is a potent activator of protein kinase C shown to have multiple, dose‐dependent effects on a range of cell‐types and cellular processes (Boyle *et al*. 2014). Of particular relevance to our results are experiments in laboratory rodents that have shown tigilanol tiglate initiates (a) an acute, short‐term, highly localised pro‐inflammatory response at the site of application that activates the patient's innate immune system (Boyle *et al*. 2014) and (b) differential expression of genes in keratinocytes (grown *in vitro*) that have well‐established roles in modulation of cell proliferation and cell migration processes (Moses *et al*. 2015).

Tigilanol tiglate was used as a topical gel in Case 1. The largest exophytic lesions would potentially have also been candidates for intradermal administration (intralesional injection), but the risk averse owners declined intralesional therapy. The effectiveness of tigilanol tiglate in this patient with PV‐induced disease is consistent with similar efficacy of this agent for treating feline viral plaques and Bowenoid SCC *in situ* (Martine Perkins and Richard Malik, unpublished observations) and equine sarcoids (Reddell and colleagues, unpublished observations). Tigilanol tiglate might also prove useful of oral squamous cell carcinomas, some of which have been recently shown to be associated with canine papillomavirus type 17 (Munday *et al*. 2016).

Other options we considered in these patients included the use of conventional surgery (e.g. *en bloc* resection of the severely affected portion of skin, followed by reconstructive surgical techniques), cryosurgery (Krahwinkel 1980) or the application of a medical laser to destroy individual plaques (Persky 1984; Knight *et al*. 2016). In terms of medical therapy, *α*‐interferon, retinoids and 5‐flourouracil could also be considered. In dogs, 1.5‐2 million units of IFN‐*α*‐2a (Roferon‐A, Hoffman‐LaRoche) given subcutaneously three times a week has been reportedly effective in the treatment of severe cases of oral and/or cutaneous viral papillomatosis (Nagata & Rosenkrantz 2013). In addition, IFN‐*α*‐2a therapy (1000 units orally once daily on a 21‐day‐on, 7‐day‐off schedule) has been reported as adjunct therapy for canine pigmented plaques (Stokking *et al*. 2004). The effectiveness of IFN‐*α*‐2b (Intron A, Schering‐Plough) administered orally at 30 units/mL has been mentioned anecdotally, but the recommended dose and frequency vary widely. Etretinate, a synthetic retinoid, was used in the past and found helpful in cases of extensive and hyperkeratotic warts in dogs; for example, extensive and hyperkeratotic canine pigmented plaque lesions were treated daily with etretinate (1 mg/kg orally once daily) (Nagata *et al*. 1995). However, etretinate was discontinued because of a narrow therapeutic index, although one could make a case for trialling topical tretoin. Because of the large number and wide anatomical distribution of lesions in patients (especially Case 1), treatment would have been laborious and time‐consuming, which goes some way to explain our approach of ‘watchful waiting’ in Cases 2 and 3, and the reluctance of the owner of Case 1 to continue tigilanol tiglate applications to additional lesions.

Pigmented plaques can be difficult to diagnose in the early stages due to their non‐specific histological features and lack of papillomaviral immunostaining. Furthermore, the clinical appearance of lesions at the early stages of infection is not especially reminiscent of papillomatosis, whereas late in the clinical course the ‘generic illness script’ is far more typical of this aetiology (Schmidt & Rikers 2007). PCR and sequence analysis using DNA extracted from shavings of the PEFF biopsy material is a valuable technique to confirm a diagnosis of this disease. Similar conclusions could be drawn from the Devon Rex cat described in the paper by Ravens *et al*. (2013), where an early case of feline papillomavirus‐induced Bowenoid *in situ* carcinoma was probably misdiagnosed, and later progressed to SCC, and ultimately a fatal outcome.

In conclusion, Case 1 represents the first reported case of pigmented plaques associated with CPV‐4 in a Hungarian Vizsla, and was an example of especially severe disease. Case 2 represents the second Vizsla likely with the same viral aetiology, while Case 3 was a similarly affected mild case but with a confirmed CPV‐4 aetiology. The clinical benefit of sequencing PCR amplicons from pigmented plaques in a unique breed allowed us to prognosticate for these patients, as pigmented plaques have been reported to transform to *in situ* and invasive carcinomas, but only for CPV‐3, and never (to date) for CPV‐4. Tigilanol tiglate would appear to be a potentially useful agent for the management of such cases, although obviously more work is required on a range of lesions in a large number of dogs.

## Source of funding

None.

## Conflicts of interest

Paul Reddell is the chief scientific officer of QBiotics which manufactures EBC‐46. The drug was provided gratis and apart from this, the authors received no financial support from the manufacturer.

## Ethical statement

The authors confirm that the ethical policies of the journal, as noted on the journal's author guidelines page, have been adhered to and the appropriate ethical review committee approval has been received.

## Contribution

NH and MS examined and treated Case 1. NN and GP examined and treated Case 2. JTM, GO and JSM provided pathological descriptions. JSM performed the PCR studies. Paul Reddell provided the topical tigilanol tiglate gel and instructions for its use. RM suggested confirmatory PCR and the use of tigilanol tiglate, facilitated the collaboration and, with NH, wrote the first draft of the paper, which was then reviewed by all the authors.
